# Investigation of Some Long Noncoding RNAs (LncRNAs) in Pediatric Inflammatory Bowel Disease (IBD): An Iranian Study

**DOI:** 10.1155/bri/8879418

**Published:** 2025-03-29

**Authors:** Fatemeh Khesali, Azizollah Yousefi, Seyyed Amir Yasin Ahmadi, Reza Nekouian

**Affiliations:** ^1^Department of Medical Biotechnology, School of Allied Medicine, Iran University of Medical Sciences, Tehran, Iran; ^2^Department of Pediatrics, Rasoul Akram Complex Clinical Research Development Center (RCRDC), School of Medicine, Iran University of Medical Sciences, Tehran, Iran; ^3^Preventive Medicine and Public Health Research Center, Psychosocial Health Research Institute, Iran University of Medical Sciences, Tehran, Iran; ^4^Pediatric Growth and Development Research Center, Institute of Endocrinology and Metabolism, Iran University of Medical Sciences, Tehran, Iran; ^5^Mehresoheila Cancer Charity, Karaj, Alborz, Iran

**Keywords:** gene expression, pediatrics, personalized medicine

## Abstract

**Introduction:** According to the importance of long noncoding RNAs (LncRNA) in the pathogenesis of inflammatory bowel disease (IBD) and also the lack of study for pediatric IBD in this regard, we investigated the expression of a selected panel of LncRNAs in Iranian pediatric cases of IBD compared to adult cases and healthy samples.

**Methods:** In this gene expression study, blood samples were taken from the three groups of pediatric IBD cases, adult IBD cases, and pediatric healthy samples (for gene expression calibration). The investigated LncRNAs were *UCA1*, *CCAT*, *IFNG-AS1*, and *CDKN2B*. Real-time PCR was used and fold changes (FCs) were reported.

**Results:** A total of 50 individuals were studied including 28 cases of pediatric IBD, 12 cases of controls, and 10 cases of adult IBD. *UCA1* showed upregulation in adult IBD (FC = 10.56, *p* = 0.007). *CCAT* showed downregulations for pediatric IBD (FC = 0.01, *p* < 0.001) and adult IBD (FC = 0.10, *p* = 0.039). *IFNG-AS1* showed downregulation in pediatric IBD (FC < 0.01, *p* < 0.001). CDKN2B showed upregulation in pediatric IBD (FC = 17.39, *p* < 0.001). The results were in contrast with the literature.

**Conclusion:** It seems that these LncRNAs may have different roles in pediatric IBD. Further studies are needed on pediatric cases of IBD.

## 1. Introduction

Long noncoding RNAs (LncRNAs) are a type of RNA defined as transcripts with a length of more than 200 nucleotides that are not translated into protein. These RNAs are involved in the regulation of gene expression, but their role and function are still not fully understood. LncRNAs are mainly localized in the nucleus and associated with chromatin, while another group of these molecules are found in the cytoplasm and even in extracellular fluids [1]. LncRNAs, like mRNAs, are transcribed by RNA polymerase II, and after transcription, capping and polyadenylation processes occur in them [2–4]. LncRNAs are generally involved in chromatin remodeling (i.e., DNA methylation and histone modification), epigenetic silencing, translational control, apoptosis, cell cycle regulation, metabolism, development, localization, and cell migration [4]. Also, their half-life is usually longer than the host mRNA. LncRNAs are more flexible to environmental changes than coding RNAs. Today, it is predicted that LncRNAs can play an important role during the process of natural selection and evolution of organisms due to their flexibility and semiconservation properties. LncRNAs play an important role in immune regulation [5].

Inflammatory bowel disease (IBD) is an inflammatory disease that refers to a group of different types of conditions that cause inflammation of the wall of the large intestine and sometimes the small intestine. IBD is a disease with a multifactorial background, which is associated with autoimmune diseases with a growing prevalence, which interacts with genetic and environmental factors. This disease is generally divided into two categories: ulcerative colitis (UC) and Crohn's disease (CD). CD can be associated with extra-colon involvement [6]. The exact cause of IBD has not been determined, but the evidence shows that UC originates from a set of microbial, genetic, immune, and lifestyle factors (such as diet) and environmental factors such as stress and air pollution. They also play a role in aggravating it [7]. In Asian countries and Iran, many people are affected by IBD. A modeling study has shown that there is an increasing trend in the prevalence of IBD in Iran [8]. IBD can also affect children. A report of pediatric IBD cases of Iran has shown that 39.0% of the cases are UC, 32.2% of the cases are CD, and 28.8% of the cases are intermediate colitis [9, 10]. In addition, pediatric IBD may have a wide-spectrum of complications such as ocular complications [11].

LncRNAs have different roles in the pathogenesis of IBD. These roles include regulation of epithelial cells, apoptosis of the intestine, lipid metabolism, cellular interactions, and regulation of regulatory T cells [12]. Genetic analysis is used to identify genomic alterations for diagnosis, assay risk, and select treatment strategies for IBD. Pediatric IBD is not an exception and genetic analysis is of great importance. For instance, Uhlig et al. suggested a monogenic panel for pediatric IBD [13]. According to the mentioned issues, two levels of genomic analysis can be considered; the discovery phase, which aims to search for new molecular targets in order to achieve a more detailed understanding of the pathogenesis of IBD, and the clinical practice, which aims to discover changes that can help manage the disease. It is clear that due to the difference in the genetic reserve of different societies and the existence of different mutations in the cases of studies that have been carried out in several countries, it is necessary to conduct a study on the genetics of the Iranian society.

Regarding the roles of LncRNAs in the pathogenesis of IBD, both *CCAT* and *UCA1* perform functions in cancers and inflammation-related disorders. According to the studies, these two LncRNAs act as sponges for *let-7* and *micro-RNA-204*, that are micro-RNAs downregulated in human colonic NCM460-NTR1 cells by NT/NTR1 signaling. *CCAT1* is the modulator of the *HOXA1* expression through sponging micro-RNA-181a-5p in multiple myeloma. Also, *IFNG-AS1* affects immune system-related genes including *IFNG*, *IL22*, and *IL26*. As well, *CDKN2B-AS1* plays a role in the regulation of tumor necrosis factor alpha (TNF-α) [12]. An animal model of acetic acid–induced UC has shown the role of *UCA1* in IBD. This LncRNA can affect micro-RNA-145 and the inflammatory cascade TLR4/NF-κB/TNF-α [14]. Another study performed in humans showed that *UCA1* could result in the progression of UC through affecting the micro-RNA-331-3p/BRD4/NF-κB pathway [15].

According to the importance of the issue and the a priori hypothesis supported by the literature [12, 14, 15], the present study was conducted to investigate the expression of a selected panel of LncRNAs in Iranian pediatric cases of IBD compared with adult cases and healthy samples.

## 2. Materials and Methods

### 2.1. Study Design

The present study was conducted with a cross-sectional design using Strengthening the Reporting of Observational Studies in Epidemiology (STROBE) and its extension for molecular epidemiology (STROBE–ME) [16]. According to PICO/PECO model, the patients/population (P) were Iran pediatric and adult cases at risk for IBD, the exposure (E) was having IBD, the comparison (C) was a comparison of the pediatric and adult cases as well as pediatric healthy cases for calibration of gene expression, and the outcome (O) was expression of LncRNAs. The investigated LncRNAs were *UCA1*, *CCAT*, *IFNG-AS1*, and *CDKN2B-AS1*. The primers are shown in Table 1. The study was performed in human participants including 28 cases of pediatric IBD, 12 cases of controls, and 10 cases of adult IBD.

### 2.2. Patients

A quota sampling was performed in Pediatric ward, Rasoul Akram Hospital, Iran University of Medical Sciences, Tehran, Iran, in 2021, in three groups: (1) pediatric IBD cases (*n* = 28), (2) adult IBD cases (*n* = 10), (3) pediatric healthy samples (*n* = 12) (for gene expression calibration). Since there was no follow-up in the present study, the temporal precedence of LncRNA expression was not authenticated, and it was considered as the outcome variable in this study. Inferential statistics was used to analyze the data and generalize the results to the reference population.

The disease was confirmed through colonoscopy and endoscopy in all patients. Then, blood was taken from the patients for detailed genetic analysis. The tests were performed on whole blood and the samples were kept in a freezer at a temperature of −80°C.

### 2.3. Gene Expression Study

The RNA was extracted from whole blood. Total cell RNA extraction was performed with the help of RNX-Plus. After RNA extraction using the TRIzol method, its quantity and quality were checked by UV spectrophotometry and agarose gel electrophoresis.

Checking the concentration of RNA by spectrophotometry is a quantitative method, and the concentration and purity of the RNA sample were obtained using optical absorption at wavelengths of 260, 280, and 230 nm (MaestroNano, Taiwan). To convert RNA to complementary DNA (cDNA), a primer is needed for hybridization with RNA, which is created by the DNA polymerase enzyme dependent on the RNA copy of cDNA. The primer used to synthesize the first strand of cDNA can be specific to the target RNA or general and binds to all RNAs. In this research, ROJE's cDNA synthesis kit (ROJE, Iran) was used. The SYBR Green Master Mix kit from Yekta Tajhiz company (Iran) was used to perform real-time PCR reactions and the Rotor-Gene^TM^ 6000 machine (Corbett company, Germany) was used. Conventional PCR reaction was performed using Taq PCR Master Mix kit (ROJE, Iran). Real-time PCR reactions were carried out in 0.1 mL vials for the Rotor-Gene 6000 machine. Negative control samples were used in all stages of gene expression analysis. First, all the materials except the cDNA template were mixed together in the microtubes, and cDNA was added at the end. All mixing steps were performed in special PCR microtubes and on ice. All real-time PCR reactions were performed using a Rotor-Gene 6000 machine and under set conditions for 40 cycles. The real-time PCR process was performed in three stages. Preincubation took place for 10 min at a temperature of 95° due to the hot start of Mastermix. Three temperature intervals of 95° for 25 s, 58° for 15 s, and 72° for 40 s were performed in 40 cycles and finally, the cooling temperature was 35° for 30 s.

### 2.4. Statistical Analysis

Considering GAPDH as the internal control, −ΔCTs were calculated. A general linear model and postestimation analysis of variance (ANOVA) were used to study the associations of the LncRNAs with study groups. All the analyses were conducted in *R 4.0.0* (R Foundation, Vienna, Austria).

### 2.5. Ethical Considerations

This project was approved by the Ethics Committee of the Iran University of Medical Sciences with registration number: IR.IUMS.REC.1399.654. All patients in the adult age category gave their written informed consent to perform the tests, and the parents of the children also gave their written informed consent.

## 3. Results

### 3.1. Primary Findings

After RNA extraction, its quantity and quality were checked by UV spectrophotometry and agarose gel electrophoresis. The presence of s18 and s28 ribosomal RNA bands were approved.

A total of 50 individuals were studied including 28 cases of pediatric IBD, 12 cases of controls, and 10 cases of adult IBD. In terms of demographic characteristics, the age range of the pediatric groups was 4–16 and the age range of adult IBD was 27–52 years. Pediatric cases consisted of 12 males, whereas adult IBD cases consisted of five males.

### 3.2. Gene Expression Study

*UCA1* was compared between the groups. This LncRNA was detected in all the samples and there were no missing data. The expression of this LncRNA was not significantly different between pediatric IBD cases and the control group (*p*=0.104). However, in adult IBD cases, a significant upregulation was observed in comparison to the control group (fold change (FC) = 10.56, *p*=0.007) (Table 2).

*CCAT* was compared between the groups. This LncRNA was detected in all the samples and there were no missing data. The expression of this LncRNA was significantly downregulated in pediatric IBD cases compared to the control group (FC = 0.01, *p* < 0.001). Also, in adult IBD cases, a significant downregulation was observed in comparison to the control group (FC = 0.10, *p*=0.039) (Table 2).

*IFNG-AS1* was compared between the groups. This LncRNA was not detected in 10 cases of pediatric IBD and in all cases of adult IBD. The expression of this LncRNA was significantly downregulated in pediatric IBD cases compared to the control group (FC < 0.01, *p* < 0.001). However, in adult IBD cases, this LncRNA was not detected (Table 2).

*CDKN2B-AS1* was compared between the groups. This LncRNA was not detected in two cases of pediatric IBD. The expression of this LncRNA was significantly upregulated in pediatric IBD cases compared to the control group (FC = 17.39, *p* < 0.001). However, in adult IBD cases, no significant change was observed in comparison to the control group (*p*=0.297) (Table 2).

### 3.3. Practical Modeling

**Figure 1:**
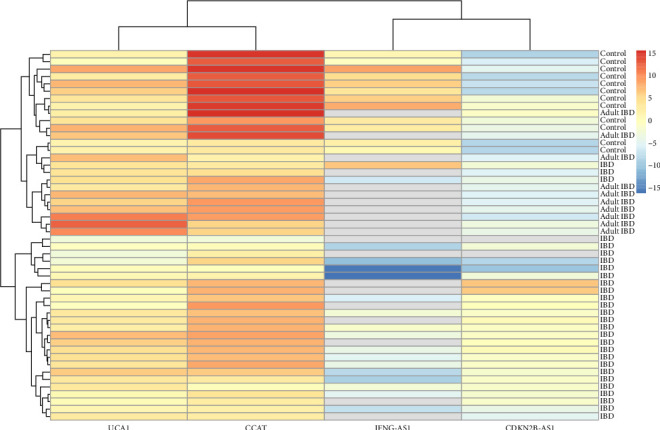
Heatmap for individual-level data of −ΔCTs. Red color indicates higher expression. Gray color indicates missing data (lack of detection).

The individual-level data of −ΔCTs is shown as a heatmap. Accordingly, most of the pediatric IBD cases were clustered in a main cluster regarding gene expression pattern (Figure 1).

**Figure 2:**
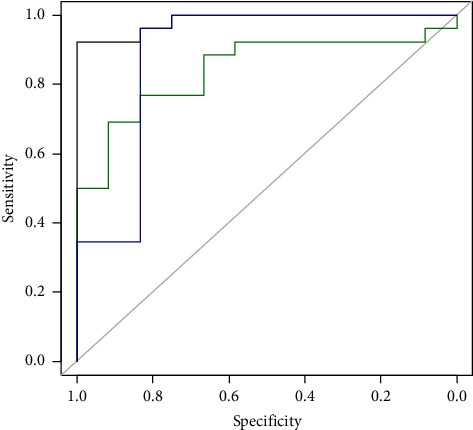
ROC curve for diagnosis of pediatric IBD for the multiple logistic regression model (black), *CCAT* (blue), and *CDNK2B* (green).

In order to use gene expression level for diagnosis of pediatric IBD from healthy children, logistic regression was considered. According to the variance inflation factors (VIFs), multicollinearity was observed as VIFs were 1.52, 2.09, 2.03, and 1.16 for *UCA1*, *CCAT*, *IFNG*, and *CDKN2B*, respectively. Therefore, CCAT and *CDKN2B* were selected for the model (*IFNG* was removed due to VIF > 2 and having many missing data, and *CCAT* was removed due to lack of significant association) (Table 3). The AUC of this model was 0.984 compared to 0.888 and 0.843 for *CCAT* alone and *CDKN2B* alone, respectively (Figure 2).

## 4. Discussion

The main aim of this study was to investigate the effects of pediatric IBD on the expression changes of selected LncRNAs compared to a pediatric control group and adult IBD. Considering the important role of the digestive system in health and its significant effects in the occurrence of various diseases in different years of life, as well as the impact of this disease in childhood and the importance of this period in the quality of life, it is very important to investigate and study this field.

According to the literature, both *UCA1* and *CCAT1* were significantly overexpressed in UC patients compared to controls. *CCAT1* and *UCA1* play an important role in the pathogenesis of inflammation-related diseases [12]. This was in contrast to the results of our study. However, the present study was focused on the pediatric cases of IBD. In the present study, *CCAT* showed significant downregulations both for pediatric and adult IBD cases. Also, no significant change in the expression of *UCA1* was observed for pediatric IBD cases. However, the significant upregulation of *UCA1* in adult IBD cases was in line with the literature. There are two hypotheses for these contradictory findings. The first one is that pediatric IBD may have a different pathogenesis from adult IBD. The second one is that the Iranian population may have different results from the viewpoint of personalized medicine.

*IFNG-AS1* acts as a potentiator of inflammation by acting as a novel regulator of interferon-gamma (IFN-γ) inflammatory responses in CD4 T-helper (Th1) cells in UC patients with high *IFNG-AS1* expression. Padua et al. found *IFNG-AS1* upregulation in adult UC individuals [17]. However, in the present study, *IFNG-AS1* was not detected in adult cases, and it was downregulated in pediatric patients compared to the controls. No study was found investigating *IFNG-AS1* in pediatric IBD. There were a lot of missing data for this LncRNA. This contradictory finding may also be for the Iranian population.

Decreased expression of *CDKN2B* increases barrier formation of colonic epithelial monolayers through disruption of claudin-2 expression. According to the study of Rankin et al., *CDKN2B* was downregulated in UC patients [18]. In the present study, there was no significant expression change for adult IBD, while a significant upregulation was found in pediatric cases of IBD compared to the controls. According to the literature [12], *CDKN2B-AS1* is upregulated in cancers and is downregulated in IBD. The results of our study were in contrast with the literature. The hypothesis is that pediatric-onset IBD may increase cancer risk in adulthood. This risk is for both gastrointestinal and extraintestinal cancers [19]. In addition, a meta-analysis approves the evidence that cancer risk in pediatric-onset IBD is higher than the general population [20]. Another meta-analysis reports this risk greater than twofolds [21]. This is why pediatric IBD has a pathogenesis similar to cancers. It is worth noting that adulthood IBD is not associated with increased cancer risk based on a meta-analysis. Although in some specific cancers significant associations were found, such associations might be due to confounding effects of smoking habits [22].

The present study had some limitations. Due to the lack of time and budget of the project, the investigation was not carried out at the protein level. However, this limitation might not affect the results as the LncRNAs are not protein-coding. Another limitation was the lack of previous studies on the pediatric IBD to investigate the reproducibility of the results. However, the results of this study can be considered as a pilot for further investigation in pediatric cases of IBD. Further research on pediatric IBD and its relationship with adulthood cancers is strongly suggested.

## 5. Conclusion

The results of the present study were in contrast with the results reported in the literature. However, the previous papers were on adult IBD cases. It seems that the studied LncRNAs may have different roles in the pathogenesis of pediatric IBD. However, peer studies should be conducted in the future to investigate the reproducibility of this evidence.

## Figures and Tables

**Table 1:** Primers.

Gene	Sequences 3′ ⟶ 5′	Primer
*CCAT1*	5′AGCAGGCAGAAAGCCGTATC-3′	F
	5′-CCCAGGTCCTAGTCTGCTTG-3′	R

*CDKN2B-AS1*	5′-TTCTAGAAGAAAACCGGGGAGAT-3′	F
	5′-TTCAATTTCCTAACCTGTTACCTCT-3′	R

*UCA1*	5′-TCAGACAAACAACCTACAACCC-3′	F
	5′-ATCCTTTTTATAGGCGGCAGG-3′	R

*IFNG-AS1*	5′-ACATGTGGGTCCAATGTGAA-3′	F
	5′-TGTTAGCAGTTGGTGGGCTT-3′	R

GAPDH	5′-GTGAACCATGAGAAGTATGACAAC-3′	F
	5′-CATGAGTCCTTCCACGATACC-3′	R

**Table 2:** Gene expression study using the general linear model.

LncRNA	Group	Expression		ANOVA	
		FC (95% CI)	*p* value	*F* (*p* value)	*R*-squared
*UCA1*	Control	Reference		11.76 (< 0.001⁣^∗^)	0.334
	Pediatric IBD	0.33 (0.08–1.27)	0.104		
	Adult IBD	10.56 (1.98–56.69)	0.007⁣^∗^		

*CCAT*	Control	Reference		15.26 (< 0.001⁣^∗^)	0.394
	Pediatric IBD	0.01 (< 0.01–0.05)	< 0.001⁣^∗^		
	Adult IBD	0.10 (0.01–0.89)	0.039⁣^∗^		

*IFNG*	Control	Reference		35.62 (< 0.001⁣^∗^)	0.560
	Pediatric IBD	< 0.01 (< 0.01–0.01)	< 0.001⁣^∗^		
	Adult IBD	0			

*CDKN2B-AS1*	Control	Reference		8.87 (0.001⁣^∗^)	0.283
	Pediatric IBD	17.39 (4.09–73.77)	< 0.001⁣^∗^		
	Adult IBD	2.53 (0.43–14.96)	0.297		

⁣^∗^Significant at *p* < 0.05.

**Table 3:** Multiple logistic regression for diagnosis of pediatric IBD.

LncRNA (−ΔCT)	Beta coefficient (SE)	*p* value
*CCAT*	−0.858 (0.360)	0.017
*CDKN2B-AS1*	0.960 (0.418)	0.022
Intercept	11.050	0.011

Abbreviation: SE, standard error.

## Data Availability

The data that support the findings of this study are available on request from the corresponding author. The data are not publicly available due to privacy or ethical restrictions.
